# Corneal Biomechanical Changes Induced by Chronic Elevated Intraocular Pressure: Implications for Glaucoma Pathogenesis and Management

**DOI:** 10.1155/joph/8826306

**Published:** 2025-11-14

**Authors:** Mengzhen Xie, Zhiyong Huang, Ke Ma, Yingping Deng, Jing Tang

**Affiliations:** ^1^Ophthalmology Department of West China Hospital, Sichuan University, Chengdu 610041, Sichuan, China; ^2^Beijing Institute of Ophthalmology, Beijing Tongren Eye Center, Beijing Tongren Hospital, Beijing Ophthalmology and Visual Sciences Key Laboratory, Capital Medical University, Beijing, China; ^3^Sichuan University, Chengdu, Sichuan, China

## Abstract

The significance and contributions of corneal biomechanics in the study of chronic elevated intraocular pressure are multifaceted. Not only does it enhance our understanding of how chronic elevated intraocular pressure affects corneal structure and function, but it also offers new perspectives for the early diagnosis of glaucoma. Analysis of the cornea's biomechanical properties enables earlier identification of high-risk glaucoma patients and potential development of personalized treatment plans, thereby improving treatment outcomes. Moreover, changes in corneal biomechanics can serve as a new metric for assessing glaucoma treatment effectiveness, providing precise clinical feedback. Continued research on the role of corneal biomechanics in chronic elevated intraocular pressure and glaucoma is crucial for improving the diagnosis, treatment, and prognosis. Understanding associations between these biomechanical changes and glaucoma development can elucidate disease mechanisms, enabling more effective treatment strategies and preventive measures. This review explores the latest research developments on corneal biomechanical changes associated with chronic elevated intraocular pressure and their impact on glaucoma management, highlighting the importance of these changes in clinical practice.

## 1. Introduction

Chronic elevated intraocular pressure (IOP) is a condition where IOP remains consistently above the normal range (typically 10–21 mmHg). While elevated IOP is a major risk factor for glaucoma, not all individuals with high IOP develop the disease [1, 2]. Glaucoma, the second leading cause of blindness globally, is characterized by elevated IOP, visual field (VF) loss, and optic nerve damage [3, 4]. Primary open-angle glaucoma (POAG), the most common subtype, includes high-tension glaucoma (HTG) with elevated IOP and normal-tension glaucoma (NTG) with normal IOP. Both forms involve progressive retinal ganglion cell (RGC) degeneration and optic nerve damage, underscoring glaucoma pathogenesis complexity beyond mechanical stress [5–8]. IOP control is a principal factor in glaucoma management [9]. Its reduction remains the only proven treatment to slow disease progression. Nevertheless, even with successful IOP control, most patients exhibit progressive vision loss [10]. Conditions causing chronic IOP elevation include POAG, secondary glaucoma, ocular hypertension, uveitis, ocular tumors, corticosteroid use, and systemic diseases like diabetes or hypertension. Distinguishing glaucoma from ocular hypertension—elevated IOP without optic nerve damage—is clinically critical due to differing management strategies. Biomechanical research highlights mechanical stress impact on ocular tissues. Professor Wang Yan's work demonstrates that mechanical stress, such as increased IOP, alters the corneal biomechanical properties by influencing corneal cell behavior and extracellular matrix (ECM) remodeling [11]. The ECM, shared across the cornea, trabecular meshwork (TM), sclera, and lamina cribrosa (LC), contributes to outflow resistance in glaucoma [12]. ECM compositions in these tissues share similar primary fibrous collagen components. Studies show corneal biomechanics not only influence IOP measurement accuracy but may also contribute to glaucoma progression via mechanical stress transmission through the scleral-optic nerve head (ONH) pathway [13]. This suggests corneal biomechanics changes might reflect optic nerve compliance in glaucomatous neuropathy and increased TM stiffness [14]. Biomechanics research provides insights into mechanical forces across scales, from organ to cellular levels [12, 15, 16]. Increasingly, researchers focus on ocular tissue biomechanics to prevent and treat eye diseases [15, 17–20].

Numerous diseases, medications, and physiological states influence corneal biomechanics. For instance, Sen, E. et al. [21] found unilateral Fuchs endothelial dystrophy patients exhibit lower corneal hysteresis (CH) and corneal resistance factor (CRF) versus unaffected eyes and controls. Systemic autoimmune diseases (e.g., ankylosing spondylitis and rheumatoid arthritis) reduce corneal stiffness versus healthy controls [22]. Prostaglandin F2*α* analogs alter cornea microstructure, reducing stiffness [23, 24]. Furthermore, pregnancy [25], diabetes [26], refractive errors [27], and glaucoma [28] all affect corneal biomechanical properties.

By 2040, the global glaucoma population is projected to reach to 111.8 million [3]. Due to highly complex pathogenesis excitotoxicity, immune mechanisms, ischemia, and oxidative stress—alongside mechanical and vascular theories—may relate to glaucoma onset, though its exact etiology remains unclear [29]. Studies show that only 50% of patients with glaucomatous VF loss receive appropriate diagnosis or treatment [30, 31]. Incorporating corneal biomechanics assessment could enhance diagnostic accuracy and timeliness.

POAG early diagnosis poses significant challenges; thus, in vivo corneal biomechanical measurement may serve as valuable biomarkers for detecting early glaucomatous damage, identifying at-risk patients, fostering diagnostic/therapeutic innovations, and improving patient outcomes and quality of life.

## 2. Molecular Networks Governing Ocular ECM and Biomechanical Regulation

The corneal stroma, constituting the cornea's principal structural component, comprises collagen fibrils, proteoglycans (PGs), and their glycosaminoglycan (GAG) chains. These collagen fibrils (predominantly Types I and V collagen) organize into parallel arrays forming lamellar structures with characteristic periodicity. Stromal PGs—including decorin, lumican, and keratocan—and their sulfated GAG side chains (keratan sulfate (KS) and chondroitin sulfate (CS)) critically maintain collagen fibrils spatial organization. This highly ordered ECM architecture enables corneal transparency through regulated light scattering [32–34]. Research demonstrates that GAGs significantly influence corneal mechanics by forming antiparallel double-stranded structures interconnecting collagen fibrils [33, 35]. The stroma also harbors functional glycoproteins (such as fibronectin and tenascin-C), essential for corneal development and morphogenesis [36]. Consistent evidence confirms that stromal compositional and structural integrity determine optical transparency, mechanical strength, and enzymatic degradation resistance [33–35]. For instance, GAGs removal markedly reduces biomechanical properties, while corneal crosslinking enhances mechanical performance via increased cross-linking [35, 37]. Post-injury, the stroma undergoes significant ECM remodeling with altered CS and KS expression [32].

The sclera, the eyeball's outer fibrous layer, consists primarily of collagen fibrils determining mechanical strength and shape. Its ECM includes collagen (mainly Type I), elastin, GAGs, and PGs [38, 39]. Collagen fibrils—the sclera's primary structural component—directly influence biomechanical through alignment patterns and cross-linking density [38]. Sparse elastin fibers and other ECM components collaboratively regulate tissue dynamics and repair [39]. Abnormal collagen alignment or cross-linking defects biomechanical homeostasis, leading to IOP abnormalities implicated in glaucoma [39].

The TM ECM comprises diverse proteins, GAGs, and ECM molecules that collectively regulate aqueous humor outflow resistance and IOP homeostasis [40, 41]. Core components include structural proteins (collagen Types IV and VI, fibronectin, laminin, thrombospondin-1), and other ECM molecules [41, 42]. These regulate TM mechanics and aqueous humor outflow through dynamic ECM remodeling and cell–matrix interactions [40, 43]. In glaucoma, excessive ECM accumulation and structural disorganization cause TM stiffening, increasing outflow resistance and elevating IOP [44].

## 3. Cornea, Sclera, and Ocular Biomechanics

**Figure 1:**
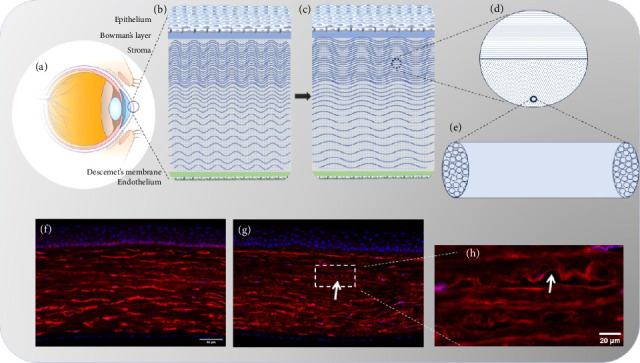
Schematic of corneal structure. (a) Diagram of the eyeball structure. (b) The five layers of the cornea. (c) Illustration of corneal collagen fiber recruitment. (d) Schematic representation of the orthogonal arrangement of central corneal collagen fibers. (e) Corneal collagen fibers are composed of collagen fibrils. (f) Immunofluorescence staining of corneal collagen fibers in a chronic high IOP rat model. (g and h) Immunofluorescence staining of corneal collagen fibers in normal rats. (Immunofluorescence staining was used to detect the distribution of Type I collagen (red), with DAPI staining to indicate cell nuclei (blue)).

The cornea is a unique anterior ocular tissue structured into five layers: epithelium, Bowman's membrane, stroma, Descemet's membrane, and endothelium (Figure 1(b)). Contributions to corneal mechanics are minimal from the epithelium, Descemet's membrane, and endothelium. While Bowman's membrane's role remains debated, the stromal layer is the primary source of corneal strength [45]. Research indicates Bowman's membrane presence minimally impacts corneal mechanical properties [46]; instead, the underlying stroma—constituting most corneal tissue—governs corneal mechanics. Human donor studies reveal the anterior 40% of stroma is the strongest region, while the posterior 60% is at least 50% weaker [47]. The stroma comprises sparse corneal cells and collagen fibrils arranged in near-parallel layers. Precise interlayer and interfibrillar spacing, maintained by PGs, is critical for transparency [48]. Native stroma contains ∼200 collagen lamellae layers (Figures 1(b) and 1(c)), whose complex arrangement ensures tissue shaping and mechanical strength [49]. The orthogonal collagen fibril organization and PG distribution preserve transparency and biomechanical properties [49].

Corneal mechanical properties are essential for IOP maintenance and eyeball shape stability. Corneal biomechanics describe morphological and structural responses to stress (e.g., IOP, trauma). In biomechanics, stress denotes force per unit area, while strain quantifies material deformation (percentage length change). Elastic modulus—the stress-strain slope—determines deformation extent under load: lower modulus materials deform more than higher modulus ones [50]. These properties critically influence corneal transparency, curvature, and visual acuity. Key parameters include stiffness, elasticity, viscosity, and viscoelasticity, which dictate force response and deformation. Tissue biomechanics depend on collagen fiber distribution in the ECM; components like elastic fibers, fibrous collagen, and GAGs achieve mechanical homeostasis. Internal collagen fiber arrangement directly governs corneal biomechanics and morphology, affecting optical properties [51]. Age, diabetes, cross-linking, and glaucoma alter collagen density and stiffness [48]. The corneal fibers are organized in a ribbon-like structure with an optimal arrangement to fulfill several functions. In the mid-layer of the cornea, some fibers are randomly dispersed to provide general resistance against IOP. In the central cornea, approximately 40% of the fibers are aligned along the superior–inferior (SI) and nasal–temporal (NT) axes, supporting eyelid movement and the action of muscles responsible for rotating the eyeball. At the limbus, the fibers exhibit a circumferential and radial organization, facilitating the transition between corneal and scleral tissues [52].

Studies have shown that elevated IOP reduces the crimping of corneal collagen fibers, which is consistent with the current theory of collagen fiber recruitment in soft tissues. This theory suggests that fibers straighten as they are stretched, leading to their recruitment and resulting in localized tissue stiffening [53, 54] (Figures 1(b) and 1(c)). Recruitment stiffens the stroma, promoting keratocyte-to-fibroblast/myofibroblast transformation [11]. Our chronic ocular hypertension rat model similarly shows decreased collagen fiber curl (Figures 1(f), 1(g), 1(h)).

Scleral biomechanical responses under elevated IOP represent a central focus in glaucoma pathology. Multiple studies highlight that the recruitment and orientation of scleral collagen fibers are critical for counteracting IOP-induced mechanical stress. Foong et al. [55] demonstrated that under physiological IOP (15 mmHg), 90% of posterior scleral collagen fibers bear mechanical loads versus one-third in cornea/peripapillary sclera, indicating regional heterogeneity. Even at pathological IOP (50 mmHg), incomplete recruitment persists, suggesting compensatory mechanisms or unresolved mechanical challenges [55]. Hua et al. [56] emphasized synergistic interaction between radially and circumferentially aligned collagen fibers in the peripapillary sclera reduces IOP-driven posterior displacement of the LC, thereby protecting the ONH from excessive deformation. The LC, a critical structure in the ONH, plays a central role in the pathological process of glaucoma. Glaucoma is characterized by progressive damage to RGC axons, and mechanical deformation and remodeling of the LC are considered one of the main causes of axonal injury. Studies have shown that elevated IOP leads to posterior displacement of the LC, disorganization of collagen fibers, and subsequent impairment of ONH blood supply and axoplasmic transport. Elevated IOP alters the viscoelasticity and anisotropy of both the sclera and the LC, which may reduce the tissue's resistance to external forces and its self-recovery ability. These findings suggest that a thinner prelaminar tissue may be associated with greater relative posterior movement of the ONH relative to the surrounding prelaminar tissue, and the LC may play an important role in preventing excessive posterior displacement of the ONH during acute IOP elevation [57].

POAG involves scleral structural alterations (especially ONH region), driving RGC degeneration. Abnormal scleral biomechanics impede IOP fluctuation compensation, exacerbating progression [58]. As the primary load-bearing tissue, changes in the mechanical properties of the sclera, especially in the peripapillary region, directly affect the mechanical environment of the ONH, causing RGC damage [59–61]. Multiple studies have demonstrated that in glaucoma patients, the sclera exhibits abnormal stiffness and ECM structure, such as disorganized collagen fiber arrangement or reduced cross-linking, which may compromise its ability to resist IOP [58, 59]. Additionally, regional differences in scleral stiffness (e.g., weaker peripapillary sclera) are associated with the rate of glaucoma progression [10, 60]. Chronic high IOP leads to an early (1-week) increase followed by a late (4-week) degradation of Type I collagen and elastin in rat sclera, along with a time-dependent increase followed by a decrease in matrix metalloproteinase 2 expression, suggesting an imbalance in ECM remodeling [62].

There is controversy among different studies regarding the direction of scleral mechanical changes in glaucoma. Some studies have found reduced scleral stiffness in glaucoma (e.g., ADAMTS10 mutation in a canine model leading to decreased collagen density) [10]. High-resolution imaging and finite element modeling have revealed that ECM structure changes (such as hierarchical arrangement of collagen fibers) occur in the glaucomatous sclera, leading to a decline in its mechanical properties [59, 63]. For example, in a rat model, the collagen fibers in the peripapillary sclera exhibit a circular arrangement, but their density and orientation are altered in glaucoma [63, 64].

While others have shown that artificially increasing scleral stiffness through chemical cross-linking can reduce tissue deformation caused by IOP [60, 65]. This contradiction may be attributed to species differences or variations in disease stages [10, 59]. Furthermore, noninvasive clinical measurements indicate that “ocular rigidity” is significantly lower in glaucoma patients compared to healthy individuals and is more strongly correlated with scleral stiffness than with corneal stiffness, further supporting the sclera as a key target in glaucoma biomechanics.

Selective enhancement of scleral stiffness through chemical or photo-cross-linking techniques can significantly reduce IOP-induced deformation. For instance, trans-scleral photo-cross-linking reduces peripapillary scleral strain by 47% [60], and genipin cross-linking of porcine sclera increases IOP due to increased resistance to aqueous humor outflow [65]. These findings suggest that active modulation of scleral mechanical properties may serve as a novel therapeutic strategy for glaucoma.

Dynamic optical coherence tomography measurements show that ocular rigidity in glaucoma patients is negatively correlated with axial length (*R* = −0.53) and strongly positively correlated with scleral stiffness parameters (SP-HC) (*R* = 0.62) [66]. However, existing studies have limitations: most animal models cannot fully simulate the chronic progression of human glaucoma [10, 61], and the long-term safety of scleral cross-linking therapies still requires validation [60].

## 4. Measurement Techniques for Corneal Biomechanics

Currently, the techniques used to assess the biomechanical properties of the cornea include axial tension testing [67], bulge tests [68], atomic force microscopy, Brillouin imaging, optical coherent elastography (OCE), supersonic shear wave imaging [69], ocular response analyzer (ORA) [70], phase-decorrelation ocular coherence tomography (PhD-OCT) [71], corneal visualization Scheimpflug technology (Corvis ST) [13, 72, 73], and other emerging technologies.

Currently, the ORA and Corvis ST are the main instruments used in clinical settings for assessing corneal biomechanics, with most clinical studies utilizing. ORA was the first instrument used clinically for in vivo measurement of corneal biomechanics. It employs a noncontact tonometer using a controlled air puff to indent the central cornea over an area of 3–6 mm. This process involves deforming the cornea through an initial applanation, concavity, and a second applanation. It assesses the biomechanical properties of the cornea by measuring changes in corneal shape and pressure information [74, 75]. The most commonly used parameters are CH and CRF. CH and CRF are viscoelastic parameters, not purely measures of viscosity or resistance, as they are time- and force-dependent. CH primarily reflects energy absorption, while CRF correlates more with resistance; both are components of viscoelastic behavior [74, 76]. These parameters are useful in distinguishing between keratoconus and healthy corneas. Additionally, ORA is considered a more accurate technique for measuring IOP, as it combines biomechanical metrics to minimize the impact of corneal thickness and intrinsic corneal properties on the IOP readings [77].

Corvis ST utilizes high-speed Scheimpflug imaging technology to record the deformation process of the eyeball when subjected to a short pulse of air. The system software analyzes this data in slow motion to provide information on the biomechanical properties of the eye, such as corneal elasticity and stiffness [73, 78]. Corvis ST represents an improvement over ORA as it directly monitors corneal deformation. Although both ORA and Corvis ST utilize similar air pulses, Corvis ST maintains consistent air pressure across different measurements, thus allowing for more accurate recording of corneal shape changes and enabling more precise assessments [77]. Corvis ST is widely used in ophthalmic clinics for assessing corneal biomechanical properties, surgical monitoring, and diagnosing ocular diseases [73, 79–81]. Additionally, Corvis ST has introduced the stress–strain index (SSI) algorithm, which is a finite element model based on predictions of corneal behavior considering the simulated effects of IOP and the Corvis ST air puff. This is the first in vivo-derived standard mechanical metric that can establish stress–strain curves for corneal tissue, used to plot overall corneal stiffness. SSI is independent of central corneal thickness (CCT) and bIOP but is significantly associated with age [82]. The introduction of BGF by Corvis ST provides a valuable addition to the armamentarium of glaucoma diagnostic tools. Its comparable diagnostic efficacy to CH, along with its noninvasive nature, positions BGF as a promising biomarker for the early detection and management of NTG. Future studies are warranted to further validate the utility of BGF in diverse patient populations and to explore its potential in combination with other diagnostic modalities [83].

Improvements in the speed and resolution of OCT imaging have made it possible to use high-speed OCT as an alternative to high-speed Scheimpflug imaging for monitoring air jet perturbations [84]. The basic principle of OCE involves applying force to the cornea, inducing tissue deformation, and then recording this deformation using OCT [85, 86]. OCE, with its high-resolution, noncontact nature, and depth-dependent analysis capabilities, has shown great potential in the research and clinical application of corneal biomechanical properties [84].

Brillouin imaging technology, similar to OCE, has been developed to more precisely describe the three-dimensional mechanical properties of living tissues. Based on the principle of Brillouin scattering, its unique feature is the ability to measure without the application of external force. This technique utilizes the inherent thermodynamic fluctuations within the medium, which cause minor changes in local density and pressure, subsequently altering the propagation mode similar to acoustic vibrations and changing the refractive index. Through these thermodynamic fluctuations, changes in the frequency of scattered light can be precisely correlated with the longitudinal modulus of a given tissue, thereby revealing the tissue's intrinsic biomechanical properties independent of applied external pressure. Although Brillouin imaging primarily provides one-dimensional (axial) biomechanical measurements along a given scan line, limiting its ability to detect nonaxial stiffness properties, its depth-dependent and noncontact measurement advantages make it of significant value in the study of the mechanical characteristics of living tissues [84, 87–89].

PhD-OCT is a nonintrusive technique based on OCT used for assessing the biomechanical properties of biological tissues. This technology relies on the principles of dynamic light scattering, which evaluates tissue characteristics by analyzing the scattering behavior of particles in a fluid [90]. PhD-OCT utilizes two spectral-domain OCT devices with different central wavelengths to capture images, allowing the system to scan a 10-mm range in less than 3 s [71, 91]. A key feature of PhD-OCT is that by analyzing the amplitude and phase decay rate of light scattered by particles undergoing Brownian motion, it can determine the attenuation constant. This attenuation constant is related to the viscosity of the tissue and is hypothesized to be inversely proportional to the degree of collagen confinement (and thus to the stiffness of the tissue). This hypothesis has been supported by experiments showing that this attenuation constant decreases after corneal cross-linking [71].

## 5. Corneal Biomechanics Changes Induced by Glaucoma and Their Potential Mechanisms

Research suggests that hardening of ocular structures, particularly the stiffening of the cornea, sclera, and LC, may be closely linked to the development of glaucoma. Increased stiffness of these structures could affect the distribution of intraocular stress and the eye's response to stress changes, thereby playing a role in the pathophysiology of glaucoma [12, 92]. The collagen fibers in the cornea and sclera are interconnected and have similar ECM components. Thus, changes in the corneal physiology of glaucoma patients could indirectly reflect changes in the compliance of the ONH and the compression and damage to the ONH [78, 93]. Although directly measuring the mechanical parameters of the LC remains challenging, assessing corneal features can indirectly predict the mechanical properties of the sclera and LC. Scleral stiffness plays a paradoxical role in glaucoma biomechanics. While a rigid sclera may reduce global ocular deformation, it can increase the transmission of mechanical stress to the LC, potentially exacerbating axonal damage. Conversely, excessive scleral compliance may lead to sustained deformation and remodeling of the lamina. This balance between stiffness and flexibility suggests that glaucomatous susceptibility arises not from isolated tissue properties but from the complex interplay between IOP, scleral biomechanics, and laminar resistance.

### 5.1. ORA-Based Analysis of Corneal Biomechanics

In patients with POAG and those with elevated IOP, CH and CRF values are significantly lower compared to the normal control group. Moreover, in patients with POAG, there is a significant correlation between CH, CRF, and the functional and structural characteristics of the eye (Table 1). This suggests that in the pathophysiology of POAG, CH and the CRF may play distinct roles [20]. A quantitative analysis of 1213 glaucomatous eyes and 1055 healthy eyes indicated that the CH values in the glaucoma group were significantly lower than those in the control group, and the CCT in the glaucoma group was also significantly lower than that in the control group [94].

Multiple studies indicate that patients with glaucoma who have low CH and low CCT exhibit more severe VF and RNFL damage [12, 72, 94–96]. Low CH indicates a relatively stiffer cornea, which means it is less effective at absorbing or dispersing stress in the anterior segment of the eye. If this stress is not adequately dispersed, it has the potential to transmit further to the ONH, possibly leading to indentation of the optic disc as well as damage and death of nerve cells [92]. The findings of Wells and other researchers support this view, reporting generally lower CH values in glaucoma patients and noting a significant correlation between CH and the compliance of the optic disc, as opposed to CCT. This suggests that greater optic nerve stiffness is associated with lower CH values [92]. Multiple studies have consistently demonstrated that a lower CH is significantly associated with an increased risk of both structural (e.g., ONH cupping) and functional (e.g., VF defects) progression in glaucoma [97, 98]. For instance, each 1-mmHg decrease in CH has been found to increase the risk of ONH surface depression by 29% and the risk of VF progression by 46% [97]. Wong et al. [99] observed anterior LC surface displacement and found that patients with low CH were more prone to structural deformation of the LC, suggesting that CH may exacerbate glaucomatous damage by affecting the eye's biomechanical buffering capacity. Xu et al.'s [97] longitudinal study further confirmed a significant association between low CH and ONH cupping as well as VF progression, but not with RNFL thinning, indicating that CH may primarily affect the LC rather than the RNFL. Therefore, low CH not only reflects the biomechanical properties of the cornea but also indicates a high sensitivity of the eye to changes in IOP, which is of significant importance in the pathophysiological research and management of glaucoma [100]. A lower CH is significantly associated with progressive posterior displacement of the anterior LC surface, further supporting the clinical value of CH as a risk factor for glaucoma progression [99].

A thin CCT is also widely recognized as an independent risk factor for glaucoma progression, particularly in POAG, where a thinner cornea is associated with a faster rate of VF loss [101]. The systematic review conducted by Agbato et al. [101] indicates that a thin CCT is not only a risk factor for the development of glaucoma but is also associated with a faster rate of VF loss. Condon and others attempted to measure the impact of CCT and CH on various indicators of glaucoma damage, finding that thinner CCT is associated with a higher cup-to-disc ratio, while lower CH is related to progressive VF damage, and that there is a correlation between CCT and CH [102]. There may exist a synergistic effect between CH and CCT. Jiménez-Santos et al. [98] found that the combined effect of low CH and thin CCT significantly increases the risk of progression in early-stage POAG, whereas high CH may partially mitigate the adverse impact of thin CCT. A multiyear prospective cohort study showed that about 25% of glaucoma patients with well-controlled IOP might experience VF progression over time, with thin corneas and low CH being major risk factors [103].

However, increasing evidence suggests that CH may have a closer correlation with the incidence, progression, and treatment response of glaucoma compared to CCT. This implies that measurements of CH could provide better predictions concerning glaucoma risk assessment and treatment efficacy, thereby underscoring the importance of considering corneal biomechanical parameters in glaucoma management [104]. Jammal et al. [105] added that CH is correlated with the rate of neuroretinal rim loss in minimum rim width at the Bruch's membrane opening and that CH has superior predictive power compared to CCT, emphasizing its role in predicting axonal injury. A prospective study by Susanna et al., targeting patients suspected of having glaucoma, showed that those eventually diagnosed with glaucoma had lower baseline CH [106]. Medeiros and others also demonstrated in a prospective study that baseline CH affects VF progression, with each decrease of 1 mmHg in CH accelerating the annual decline in VF index by 0.25% [107]. Statistical analysis indicates that CH is the only multifactorial variable independently associated with glaucoma, suggesting that CH plays a discriminative role in distinguishing between patients with POAG and those merely presenting with symptoms of high IOP [108]. These findings underscore the significant role of CH in the pathophysiological mechanisms of glaucoma, highlighting its potential as an indicator for assessing the risk and progression of the disease [20, 108, 109].

Additionally, corneal stiffness parameters and asymmetric CH (interocular differences) have also been shown to predict glaucoma progression [72, 110], expanding the clinical application value of traditional biomechanical indicators. Overall, compared to healthy individuals, patients with glaucoma have lower CH, and lower CH is associated with an increased risk of disease progression; however, the causal relationship remains to be proven [111].

### 5.2. Unveiling Corneal Biomechanics With Corvis ST

In studies using Corvis ST to examine corneal biomechanics, reports have indicated that corneal deformation in glaucoma patients is significantly lower than in control groups [79, 80, 112, 113]. A meta-analysis, which included six prospective studies, compared the Corvis ST biomechanical parameters between HTG patients and normal control groups. The results suggested that compared to healthy controls, HTG patients exhibit a higher degree of corneal stiffness [114]. Treatment-naïve POAG patients exhibit elevated corneal stiffness parameters (e.g., SP-A1 and GBF values), indicating that intrinsic structural stiffening of corneal tissue may correlate with the progression of glaucomatous optic neuropathy [72, 83]. SP-A1, widely recognized as a core biomarker of corneal stiffness, is markedly elevated in POAG and associated with structural alterations in the ONH (e.g., LC curvature) even in NTG [72]. Additionally, deformation amplitude (DA)—a measure of corneal biomechanical compliance—is typically reduced in glaucomatous eyes, potentially linked to elevated IOP or corneal tissue sclerosis [83, 115]. Indian researchers Pradhan et al. further demonstrated a negative correlation between DA and glaucoma severity [115].

Conversely, other studies have shown that corneal deformation in glaucoma patients exceeds that in control groups [116]. A study aimed to evaluate the dependency of biomechanical indices on IOP found that most dynamic corneal response parameters provided by Corvis ST are correlated with IOP. This research highlights the complex interplay between corneal biomechanics and IOP, indicating that various corneal biomechanical indices are influenced by changes in IOP [117]. IOP is a major confounding factor, with higher pressures exhibiting stiffer behavior; thus, the effect of IOP must be considered when determining corneal biomechanics [118, 119]. The findings suggest that corneal deformability may vary across different glaucoma subtypes and that factors such as CCT and IOP play crucial roles in determining corneal mechanical properties [120]. These insights could have important implications for the diagnosis, monitoring, and management of glaucoma, particularly in understanding the biomechanical aspects of the disease [120]. After adjusting for IOP, there were no differences in corneal biomechanical parameters measured with Corvis ST between POAG eyes and healthy control eyes [121, 122]. But several studies have indicated that, compared to healthy eyes with similar age, IOP, and axial length, eyes with glaucoma tend to exhibit lower ocular rigidity [28]. One study comparing the biomechanical properties of normal eyes, untreated POAG eyes, and treated POAG eyes found that the corneal SSI was significantly higher in untreated POAG eyes than in normal eyes. This difference remained statistically significant even after adjusting for IOP, suggesting that the corneal material or structure itself in untreated POAG eyes displays abnormal stiffness characteristics [83].

Qassim A and others have suggested that eyes with higher corneal stiffness parameters experience faster thinning of the RNFL and are more susceptible to progressive VF loss [72]. Elevated SP-A1 levels, indicative of stiffer corneas, are associated with accelerated rates of RNFL thinning [72]. In glaucoma suspects, higher corneal SSI values and lower CCT suggest thinner and biomechanically stiffer corneas, conferring a greater risk of disease progression. Corneal SSI parameters appear to synergistically interact with CCT as composite risk factors for glaucoma progression [72]. Qassim et al. [72] demonstrated that corneal stiffness parameters (e.g., SP-A1) independently predict structural and functional progression in glaucoma suspects, highlighting that corneal biomechanical properties may hold greater clinical potential than CCT alone. However, other researchers argue that stiffer eyes inflict less damage on RGCs [123, 124]. Despite numerous studies addressing the relationship between corneal biomechanics and the severity of glaucoma, conclusions still remain controversial [13, 72, 73, 78–80, 123, 125]. This discrepancy may be related to racial differences, study designs, or the effects of certain medications. These findings further emphasize the importance of focusing on the biomechanical properties of intraocular structures in understanding and treating glaucoma. Consequently, more and more researchers have begun to study the relationship between corneal biomechanics and the severity of glaucoma [72, 73].

### 5.3. Deciphering Corneal Biomechanics in NTG

Notably, the corneas of NTG patients are more deformable than normal controls, whereas the corneas of HTG and ocular hypertension patients are stiffer [126]. It is noteworthy that clinical studies measuring corneal biomechanics using ORA and Corvis ST have shown that patients with NTG have softer corneas compared to patients with HTG and the control group, which is associated with thinner CCT [73, 120, 126, 127]. Additionally, NTG eyes exhibit a faster rate of corneal inward applanation compared to normal control eyes, indicating that the cornea is more prone to deformation [128]. Corneas in NTG patients exhibit heightened deformability, characterized by lower A1Area and higher deformation amplitude, which are significantly associated with VF progression [129]. For instance, A1Area serves as an independent risk factor for NTG progression (AUC = 0.813), demonstrating its predictive value in disease advancement [129]. Compared to healthy controls, the NTG group exhibited a lower PachySlope (a parameter describing the rate of corneal thickness change from center to periphery) and a statistically significant shortening of the HC time (highest concavity time), suggesting reduced corneal viscoelastic capacity in NTG eyes [130]. This may suggest differing biomechanical properties of the eyes in NTG and HTG. Nonetheless, there are still inconsistent findings, such as those by Yang et al., who compared CH values between HTG and NTG patients and found no significant differences between them [131].

In recent years, corneal biomechanics have received widespread attention in the study of the pathogenesis of glaucoma, as changes in the biomechanical properties of the cornea are thought to be closely linked to the development of glaucoma. Some researchers have found that compared to healthy controls, glaucoma patients exhibit greater ocular stiffness [78, 132–134]. However, Kazemi and others propose that compared to healthy eyes matched for age, IOP, and axial length, eyes with glaucoma often show lower ocular stiffness [28]. Interestingly, Leung and others did not observe any statistical differences in corneal biomechanics between POAG patients and controls in their study. Corneal biomechanics play a crucial role in the study of chronic elevated IOP and contribute significantly to the understanding of ocular diseases, especially the development of glaucoma.

## 6. Summary and Outlook

Changes in corneal biomechanics induced by chronic elevated IOP represent a complex yet promising area of research that is crucial for enhancing the accuracy of glaucoma diagnosis and treatment effectiveness. The variability in current research findings may be partly due to the limited accuracy and repeatability of corneal biomechanical measurements. Measurements from different corneal biomechanical assessment devices (e.g., Corvis ST and ORA) exhibit weak interdevice correlations, suggesting limited comparability of biomechanical parameters across platforms. The currently more mainstream perspective is that a thinner, more elastic, more compliant (or having greater compliance), and less rigid cornea is associated with an increased risk of glaucoma development and progression [135]. With advancements in measurement technologies and a deeper understanding of the biomechanical mechanisms of glaucoma, research on corneal biomechanics will continue to provide new insights and strategies for the management of glaucoma. Understanding changes in the corneal biomechanics of patients with POAG is significant for glaucoma management; it aids in improving the accuracy of IOP measurements. Furthermore, changes in corneal biomechanical parameters may become new indicators for monitoring the progression and treatment response of glaucoma.

## Figures and Tables

**Table 1:** Summary of key corneal biomechanical parameters and their association with glaucoma progression.

Parameter	Brief description	Implication for glaucoma pathogenesis	Potential clinical utility in management
CH [12, 72, 94–98].	A biomechanical metric reflecting the cornea's viscous damping capacity to absorb and dissipate energy.	Reduced CH correlates with worse VF and RNFL damage. Reduced CH increases LC strain, promoting its remodeling and posterior displacement.	Strong prognostic factor: Lower CH independently predicts structural/functional progression. Each 1-mmHg decrease raises VF progression risk by 46%. Treatment guidance: Indicates need for aggressive therapy and lower target IOP. Synergistic with CCT for risk stratification.

CCT [101, 102].	A static anatomical measurement of the corneal thickness at its center.	Thinner CCT is a proven risk factor for POAG progression, associated with faster VF loss and higher cup-to-disc ratio.	Risk stratification: Independent glaucoma progression risk factor; warrants closer monitoring in thin CCT patients. Treatment decision: May influence the target IOP setting, often combined with CH.

SSI [72, 83]	A parameter that reflects the intrinsic material stiffness of the cornea, independent of its CCT.	Elevated SSI indicates corneal stiffening in POAG, altering force transmission to the optic nerve and increasing susceptibility to damage.	Prognostic indicator: Elevated SSI with thin CCT indicates high progression risk; complements CH/CCT in risk assessment.

SP-A1 [72]	A core biomarker representing the intrinsic material stiffness of the cornea at the first applanation event.	Elevated in POAG/NTG, indicating corneal stiffening; directly linked to accelerated RNFL thinning and ONH structural changes.	Prognosticator: Independently predicts structural/functional progression; superior predictive value versus CCT alone.

BGF [83]	A composite index derived from dynamic corneal response parameters to estimate overall corneal biomechanical behavior and susceptibility.	Elevated BGF indicates corneal stiffening, correlating with glaucomatous neuropathy progression.	Early detection & diagnosis: Noninvasive biomarker with CH-comparable efficacy; valuable for NTG. Enhances risk stratification in combined modalities

## Data Availability

The data that support the findings of this study are available from the corresponding authors upon reasonable request.
